# Exploring the Linkages Between Non-Communicable Disease Multimorbidity, Health Care Utilization and Expenditure Among Aboriginal Older Adult Population in India

**DOI:** 10.3389/ijph.2022.1604333

**Published:** 2022-03-07

**Authors:** Parul Puri, Sanghamitra Pati

**Affiliations:** ^1^ International Institute for Population Sciences (IIPS), Mumbai, India; ^2^ Regional Medical Research Center (ICMR), Bhubaneswar, India

**Keywords:** multimorbidity, health care, India, aboriginal, tribal, expenditure

## Abstract

**Objective:** The study investigates the magnitude and correlates of non-communicable disease multimorbidity and explores its linkages with health care utilization and out-of-pocket expenditure among aboriginal or tribal older adults.

**Methods:** The study employed data on 11,365 older adults from Scheduled Tribes from the Longitudinal Ageing Study in India, 2017-18. A disease score was computed integrating sixteen non-communicable diseases. Descriptive, bivariate, and multivariable analyses were performed to identify the magnitude and correlates of multimorbidity. The study further explored the linkages between selected diseases and multimorbidity with health care utilization and expenditure.

**Results:** The findings suggest that 27.1 and 14.5% of the aboriginal population lived with single or multiple disease, respectively. Hypertension and gastrointestinal disorders were frequent diseases. Higher age, Muslim religion, higher education, unemployment, and affluent background were the major correlates of multimorbidity. Health care utilization, mean expenditure on hospitalization, and outpatient visits increased significantly with multimorbidity.

**Conclusion:** Multimorbidity is emerging as a health care challenge among the aboriginal population. Measures need to be taken to assess the multimorbidity burden and reduce health care expenditure, ensuring health equity among country’s vulnerable population.

## Introduction

The United Nations adopted Sustainable Development Goals (SDG), with the primary aim to ensure peace and prosperity for all by 2030 (1). To achieve this, there is a need to foster a balance between global social, economic and environmental needs. In this regard, the SDG adopted 17 goals, 169 targets, and 231 unique indicators to measure the progress achieved till 2030 (1). Considering the social prerequisites, SGD’s 10th goal endorses reducing inequalities among and within countries (1). Inequalities within societies place a dual burden, firstly, by inhibiting the speed of development and secondly, by weakening solidarity between the community and hampering the relationship between citizens and government (1, 2). This often leads to unequal access to opportunities and impedes political and economic decision-making, thus, preventing vulnerable communities from leading socially and economically productive lives (2). Goal ten of the SDGs adopted appeals to all nations to reduce economic inequalities and other discrimination based on origin, race, ethnicity, age, sex, disability, or religion; and promote social, economic, and political inclusion for all (2). United Nations recognizes these inequalities in opportunities and developmental variations within communities as decisive encumbrances in achieving global goals (2).

India is a large and diverse country (3); which when combined with the rigid social stratification, it acts as the root cause for the social and economic inequalities, which in turn, reciprocates in the form of disparities in opportunities and political and economic decision-making (2). The Aboriginal population, which comprises around 750 tribes, and account for 8.6 percent of the Indian population, is considered the most vulnerable sub-group in the country (2). The government of India has employed several initiatives, including scholarships for education and various entrepreneurship and livelihood opportunities for aboriginal communities in the country (2). Despite these initiatives, formulating policies and programs for the most disadvantaged population remains a challenge, majorly because of the lack of empirical evidence (2). Thus, Aboriginals commonly referred to as the tribal population, still experience social, political, and economic inequity affecting their accessibility and affordability to the fundamental right to a healthy life (2)**.**

Existing evidence highlights the preponderance of adverse health outcomes among the aboriginal population compared to their counterparts (4), these include higher communicable disease burden and mortality rates (5, 6), poor nutritional outcomes (5, 7), poor sanitation (5), a higher burden of risky health behaviours (5, 8), and poor access to health care (4, 5). Also, these studies are either based on smaller samples or specific regions in the country (9, 10). These studies do not incorporate the growing burden of non-communicable disease among the aboriginal population. Despite the work done, there is an urgent need to produce empirical evidence that captures a detailed non-communicable disease multimorbidity profile for the aboriginal population at the national level. Given their prevailing socio-economic disadvantage, the implications posed by multimorbidity on the aboriginal population are more severe than that impinged on their counterparts. Thus, the study aims to estimate the burden of non-communicable disease multimorbidity and its correlates among India’s aboriginal older adult population. Furthermore, it explores the linkages of multimorbidity with healthcare utilization and expenditure incurred during hospitalization and outpatient visits among these populations.

## Methods

### Data and Sample Size

The study employed the first wave of secondary data from the Longitudinal Ageing Study in India (LASI), 2017–18. LASI is affiliated with the International Institute for Population Sciences (IIPS), Harvard T. H. Chan School of Public Health, and the University of Southern California; and was launched under the stewardship of the Ministry of Health and Family Welfare, Government of India (11). LASI wave-1 provides nationally representative information on the social, economic, and health correlates for the older adult population from 35 states and union territories in India (except Sikkim). Thus, LASI included information on the individuals aged 45 years and above and their spouses residing in the same household, irrespective of age (11). A multi-stage stratified areas probability cluster sampling design, was implemented in LASI. Considering three and four stages in the rural and urban areas, respectively. A detailed description of the sampling design can be seen elsewhere (11).

The present study utilized information from the individual dataset, which contained 72,250 individuals (18 years and older population) (11). From this, we retained information on 65,562 individuals aged 45 years and older. Out of these 65,562 samples, we derived information on 11,365 Scheduled Tribes (aboriginal) older adult population for the final analysis.

### Measures

The study included information on socioeconomic and demographic variables available in LASI. Three outcome variables, 1) the number of non-communicable morbidities, 2) health care utilization, and 3) out-of-pocket expenditure, were employed to identify the correlates and impact of multimorbidity among aboriginal older adult population in the country. Non-communicable multimorbidity (or multimorbidity) was defined as the simultaneous occurrence of two or more non-communicable diseases. A detailed description of the study measures is presented in Table 1.

**TABLE 1:** Description of the variables included in the study, Longitudinal Ageing Study in India (LASI), 2017–18.

Independent variables	Categories
Age (in years)	45–49 years; 50–54 years; 55–59 years; 60–64 years; 65–69 years; 70–74 years; 75–79 years; 80 years and older
Sex	Male; Female
Place of Residence	Urban; Rural
Religion	Hindu; Muslim; Christian; Others
Level of education	No education; Up to Primary, Middle school completed, Higher Secondary and above
Occupation	Unemployed; Blue Collar; White Collar, Pink Collar; Not Classified/Others
Wealth	Poor; Non-poor
**Outcome 1:** Number of non-communicable diseases (morbidities)	

Question	Categories
Has any health professional ever diagnosed you with the following chronic conditions or diseases?	1. No morbidity: individuals with no chronic NCDs 2. Single morbidity: individual with exactly one NCD 3. Multimorbidity: simultaneous occurrence of two or more NCD
1. Asthma (Yes/No)	
2. Cancer (Yes/No)	
3. Chronic bronchitis (Yes/No)	
4. Chronic renal failure (Yes/No	
5. Chronic obstructive pulmonary disease (Yes/No)	
6. Diabetes mellitus (Yes/No)	
7. Gastrointestinal disorder (Yes/No)	
8. Chronic heart disease (Yes/No)	
9. High cholesterol (Yes/No)	
10. Hypertension (Yes/No)	
11. Musculoskeletal disorders (Yes/No)	
12. Neurological and psychiatric disorder (Yes/No)	
13. Skin disease (Yes/No)	
14. Stroke (Yes/No)	
15. Thyroid disease (Yes/No)	
16. Urinary incontinence (Yes/No)	
**Outcome 2:** Indicators of Health Care Utilization	

Indicators	Question	Categories
Visited Health Facility/Health care Providers	In the past 12 months, have you visited any health care facility or any health professional has visited you?	No
		Yes
Number of inpatient hospitalizations	Over the last 12 months, how many times you were admitted as patient to a hospital/long term care facility for at least one night?	Never
		One or more (≥1)
Duration of stay in the hospital	How many nights have you spent in the hospital during the a past 12 months?	Never
		1–3
		Four or more
Number of outpatient visit	In past 12 months, how many times did you receive healthcare or consultation from a healthcare provider (including home visits)	Never
		One or more (≥1)
**Outcome 3:** Out of Pocket Expenditure (OOPE)		
**Type of Expenditure**	**Question**	**Categories**
Expenditure on Hospitalization in last 12 months	In your recent visits, how much did you or your household pay for	
	1. Health care provider’s fees (consultation charges)	1. Physician’s fees
	2. Medicines form hospital	2. Medicines
	3. Medicines from outside.	3. Laboratory tests
	4. Tests/investigations
	5. Hospital and nursing home charges including bed charges and related expenses	4. Hospital Charges
	6. Operation theater charges, surgery charges and related expenses	5. Surgery Charges
	7. Blood, oxygen cylinder	6. Blood, oxygen cylinder
	8. Transportation	7. Transportation
	9. Expenses of the accompanying person(s) (food/accommodation)	8. Accommodation/food
	10. Expenditure not elsewhere reported (others)	9. Other Expenditure
Expenditure on last outpatient visit	In your last visit how much, you or your household pays for	
	1. Health care provider’s fees (consultation charges	1. Physician’s fees
	2. Medicines form hospital	2. Medicines
	3. Medicines from outside	3. Laboratory tests
	4. Tests/investigations	4. Hospital Charges
	5. Hospital and nursing home charges including food charges	5. Surgery Charges
	6. Operation theater charges, surgery charges and related expenses	6. Blood, oxygen cylinder
	7. Blood, oxygen cylinder	7. Transportation
	8. Transportation	8. Accommodation/food
	9. Expenses of the accompanying person(s) (food/accommodation)	9. Other Expenditure
	10. Expenditure not elsewhere reported (others)	

Sources of financing	Question	Computation Procedure
Sources of financing incurred expenditure on hospitalization in past 12 months	What are the sources through which you met the expenses for health care and what is the amount covered?	Reimbursed Amount = Insurance coverage + Reimbursement from employer
	1. Personal Income	
	2. Household income excluding personal income	
	3. Savings	
	4. Loans (bank/friends/relatives)	
	5. Contribution from friend/relatives	
	6. Selling assets/property	
	7. Insurance coverage	
	8. Reimbursement from employer	
	9. Others	
Sources of financing incurred expenditure on outpatient visits	What are the sources through which you met the expenses for health care and what is the amount covered?	Reimbursed Amount = Reimbursed from insurance coverage + Reimbursement from employer
	1. Personal Income	
	2. Household income excluding personal income	
	3. Savings	
	4. Loans (bank/friends/relatives)	
	5. Contribution from friend/relatives	
	6. Selling assets/property	
	7. Insurance coverage	
	8. Reimbursement from employer	
	9. Others	

The Out-of-pocket Expenditure (OOPE) was defined as the total amount spent on (A) hospitalization and (B) outpatient visits net of compensation received in the form of reimbursement from insurance or employer (12).

### Statistical Analysis

The burden of selected NCDs and multimorbidity were examined using prevalence rates for the study population (13). The background profile of the respondents was explored, along with their bivariate associations with single disease and multiple diseases, respectively. Further, the multinomial logistic regression model was fitted, and Relative Risk Ratios (RRR) and 95% confidence intervals (CI) were estimated to identify the correlates of multimorbidity.

To assess the implications of multimorbidity, the pattern of healthcare utilization was studied for selected diseases and the number of NCDs. Furthermore, the association between disease condition and healthcare utilization was explored using chi-square and Fisher exact test’ *p*-values. Variation in the OOPE for the selected NCDs and number of morbidities were evaluated using mean expenditure. As the information supplied on the OOPE was skewed, a Kruskal Wallis’ Chi-square test was used to assess the significant difference between mean expenditure for individuals living with single or multimorbidity. Furthermore, the distribution of OOPE on hospitalization and outpatient visits was visualized using a donut chart, which illustrates what part of the total OOPE was contributed by specific items during hospitalization and outpatient visits, respectively.

All statistical analysis was performed using STATA 15.0 (StataCorp™, College Station, Texas) and RStudio 1.1.463 (R Studio, Inc.). All estimates presented are derived by applying suitable sampling weights (individual weights at national level) supplied by LASI (11).

## Results

The study is based on 11,365 aboriginal individuals aged 45 years and above from LASI, wave-1, 2017-18. Table 2 illustrates the disease profile among India’s aboriginal older adult population. The findings suggest that hypertension (15.1%), gastrointestinal disorders (14.7%), musculoskeletal disorders (10.0%), diabetes (5.1%), and skin diseases (4.6%) were the most prevalent NCDs among the aboriginal older adult population in the country. In addition, 27.1 percent of the individuals were living with single morbidity, whereas 14.5 percent were reported living with multimorbidity.

**TABLE 2:** Disease profile of the aboriginal older adult population, Longitudinal Ageing Study in India (LASI), 2017–18.

Diseases	Frequency	Prevalence (per 100 individual)
Asthma	303	3.74
Cancer	52	0.38
Chronic bronchitis	45	0.44
Chronic heart disease	165	1.18
Chronic obstructive pulmonary disorder	86	0.86
Chronic renal disease	104	0.53
Diabetes	830	5.05
Gastrointestinal disorder	1,643	14.72
High cholesterol	245	0.71
Hypertension	2,287	15.31
Musculoskeletal disorders	964	10.01
Neurological and psychological disorder	162	1.54
Skin disease	386	4.59
Stroke	161	1.19
Thyroid disorder	161	1.35
Urinary incontinence	204	2.41
Single morbidity	2,934	27.08
Multimorbidity	1922	14.53

Table 3 describes the study sample. The study sample comprised 19.1 percent of the individuals from 45 to 49 years. Around forty-five percent of the aboriginal population were males, and 88 percent lived in rural areas. Eighty-one percent of them followed the Hindu religion. Around sixty-seven percent of the aboriginal individuals received no education, and nearly 38 percent were unemployed. Fifty-nine percent of the aboriginal population were poor, and seventy-three percent were currently in marital union.

**TABLE 3:** Descriptive, bivariate and multivariable results for aboriginal older adult population, Longitudinal Ageing Study in India (LASI), 2017-18.

Background characteristics	Weighted percentages (frequency)	Bivariate analysis prevalence (per 100 population)		Relative risk ratio (95% C.I.)	
		Single morbidity	Multimorbidity	Single morbidity vs. No morbidity	Multimorbidity vs. No morbidity
Age (in years)					
45–49 (Ref.)	19.14 (2,429)	25.49 (25.45, 25.53)	10.71 (10.68, 10.74)	1.00	1.00
50–54	18.18 (1980)	25.28 (25.23, 25.32)	11.34 (11.31, 11.38)	1.04 (0.78, 1.39)	1.22 (0.86, 1.69)
55–59	14.97 (1783)	25.29 (25.24, 25.34)	14.06 (14.02, 14.10)	1.12 (0.83, 1.51)	1.53** (1.02, 2.30)
60–64	15.56 (1726)	27.24 (27.19, 27.29)	16.19 (16.15, 16.24)	1.32 (0.99, 1.75)	1.97*** (1.36, 2.85)
65–69	14.44 (1,449)	29.32 (29.27, 29.37)	14.65 (14.61, 14.69)	1.42 (0.99, 2.02)	1.61** (1.08, 2.04)
70–74	8.43 (883)	30.44 (30.37, 30.51)	19.33 (19.27, 19.39)	1.57** (1.05, 2.35)	2.10*** (1.31, 3.37)
75–79	5.08 (528)	28.39 (28.31, 28.48)	27.21 (27.13, 27.30)	1.65** (1.04, 2.63)	3.17*** (1.74, 5.78)
80 years or above	3.94 (587)	32.09 (31.99, 32.19)	16.08 (16.00, 16.16)	1.69** (1.06, 2.69)	1.63* (1.01, 2.86)
		χ^2^ *p*-value < 0.001			
Sex					
Male (Ref.)	45.31 (5,256)	26.47 (26.44, 26.49)	15.82 (15.80, 15.85)	1.00	1.00
Female	54.69 (6,109)	27.57 (27.55, 27.60)	13.45 (13.43, 13.47)	1.12 (0.89, 1.40)	0.85 (0.66, 1.09)
		χ^2^ *p*-value < 0.001			
Residence					
Rural (Ref.)	88.09 (8,801)	26.75 (26.73, 26.77)	13.63 (13.62, 13.65)	1.00	1.00
Urban	11.91 (2,564)	29.44 (29.39, 29.51)	21.12 (21.07, 21.17)	1.21 (0.84, 1.40)	1.36 (0.91, 2.03)
		χ^2^ *p*-value < 0.001			
Religion					
Hindu (Ref.)	80.64 (4,958)	26.09 (26.06, 26.11)	14.23 (14.21, 14.24)	1.00	1.00
Muslim	1.95 (1,207)	49.79 (49.63, 49.94)	20.44 (20.31, 20.56)	3.47*** (1.40, 8.63)	2.17** (1.12, 4.21)
Christian	13.60 (4,715)	29.85 (29.80, 29.90)	15.24 (15.20, 15.28)	1.21 (0.99, 1.48)	1.06 (0.82, 1.37)
Others	3.82 (485)	26.41 (26.32, 26.51)	15.27 (15.19, 15.35)	1.03 (0.61, 1.75)	1.06 (0.51, 2.17)
		χ^2^ *p*-value < 0.001			
Level of education					
No Education (Ref.)	67.07 (6,206)	26.25 (26.22, 26.27)	12.08 (12.06, 12.10)	1.00	1.00
Up to primary	18.99 (2,942)	28.36 (28.31, 28.40)	19.15 (19.11, 19.18)	1.33** (1.01, 1.75)	1.81*** (1.36, 2.41)
Middle school completed	10.86 (1,633)	28.10 (29 = 8.03, 28.16)	20.17 (20.11, 20.22)	1.37 (0.97, 1.94)	1.80*** 91.21, 2.65)
Higher secondary and above	4.38 (584)	32.33 (32.23, 32.44)	19.78 (19.69, 19.87)	1.75 (0.87, 3.49)	1.61 (0.92, 2.78)
		χ^2^ *p*-value < 0.001			
Occupation					
Unemployed (Ref.)	37.60 (4,784)	28.30 (28.26, 28.33)	20.06 (20.03, 20.09)	1.00	1.00
Blue collar	48.53 (3,819)	26.36 (26.33, 26.39)	10.48 (10.46, 10.50)	0.87 (0.69, 1.09)	0.48*** 90.36, 0.64)
White collar	1.78 (387)	26.51 (26.37, 26.65)	22.54 (22.41, 22.67)	0.72 (0.37, 1.38)	0.75 (0.40, 1.40)
Pink collar	2.02 (256)	21.00 (20.87, 21.12)	14.03 (13.92, 14.14)	0.56 (0.31, 1.01)	0.45** (0.23, 0.89)
Not classified/others	10.07 (2,119)	27.26 (27.20, 27.32)	12.03 (11.99, 12.08)	0.91 (0.67, 1.25)	0.54*** (0.36, 0.79)
		χ^2^ *p*-value < 0.001			
Wealth					
Poor (Ref.)	58.55 (5,712)	26.67 (26.63, 26.68)	11.00 (10.99, 11.02)	1.00	1.00
Non-poor	41.45 (5,653)	27.66 (27.63, 27.69)	19.50 (19.47, 19.52)	1.13 (0.92, 1.37)	1.89*** (0.50, 2.39)
		χ^2^ *p*-value < 0.001			
Marital status					
Currently in union (Ref.)	72.80 (8,349)	27.39 (27.37, 27.42)	14.40 (14.38, 14.42)	1.00	1.00
Not in union	27.20 (3,016)	26.21 (26.18, 26.25)	14.85 (14.82, 14.88)	0.82 (0.65, 1.04)	0.84 (0.63, 1.12)
		χ^2^ *p*-value < 0.001			
Total	100.00 (11365)	27.08 (27.05, 27.09)	14.53 (14.51, 14.54)		

Note. (Ref.)-Reference category for multivariable multinomial logistic regression analysis.

*p < 0.05, **p < 0.01, ***p < 0.001.

Considering the findings from bivariate analysis from Table 3, twenty-seven percent of the aboriginal individuals reported living with single morbidity, whereas around 15 percent were living with multimorbidity. The percent living with single disease was highest for respondents aged 80 years and above (32.1%). Moreover, the burden of single disease was highest for females (27.6%) and respondents belonging to urban areas (29.4%) and from the Muslims (49.4%) religion. The percent of individuals living with multimorbidity were highest for aboriginal individuals in the age range 75–79 years (27.2%), males (PR = 15.8%), urban residents (21.12%), Muslims (20.4%), had a white-collar job (22.5%), belonged to non-poor (19.5%) wealth category and were currently not in union (14.9%).

Table 3 further presents the findings from multinomial logistic regression considering ‘no morbidity’ as the base outcome. The findings suggest that age, religion, level of education, occupation, and wealth were significantly associated with multimorbidity among the aboriginal older adult population. The likelihood of reporting multimorbidity was higher among respondents in higher age groups than individuals aged 45–49 years. The likelihood of reporting multimorbidity was higher among the respondent following the Muslim [RRR = 2.17] faith than the Hindu respondents. The likelihood of reporting multimorbidity increased with the education of the respondent. The likelihood of reporting multimorbidity was higher for individuals in the non-poor wealth category [RRR = 1.89] than poor individuals. Respondents who had a blue-collar job [RRR = 0.48] were less likely to be affected with multimorbidity than unemployed individuals. Similarly, respondents having a pink-collar job [RRR = 0.45] were less likely to be affected with multimorbidity than unemployed individuals.

Table 4 presents the association between selected NCDs and four indicators of healthcare utilization, namely visited health facility or healthcare provider, number of inpatient hospitalizations, duration of stay, and number of outpatients visit. All the diseases except chronic renal disease increased the percent visit to a health facility or healthcare provider. Thyroid disorder (86.3%), neurological and psychological disorders (86.9%), cancer (82.8%), urinary incontinence (79.3%) and diabetes (74.8%) had the highest percent of the visit to a healthcare facility. The percent the visits were highest for individuals were living with multimorbidity (81.5%) compared with those living with single morbidity (68.0%).

**TABLE 4:** Association between the selected diseases and indicators of healthcare utilization for aboriginal older adult population, Longitudinal Ageing Study in India (LASI), 2017–18.

Diseases	Visited health facility/Health care providers [% (N)]			Number of inpatient hospitalization [% (N)]			Duration of stay in the hospital [% (N)]				Number of outpatient visit [% (N)]		
	No	Yes	*p*-value	Never	≥1	*p*-value	Never visited	One to three	4 or more days	*p*-value	Never	≥1	*p*-value
Asthma	40.4 (27)	59.6 (70)	0.000	88.86 (83)	11.14 (14)	0.000	88.86 (83)	6.68 (10)	4.46 (4)	0.000	50.41 (48)	49.59 (49)	0.000
Cancer	17.2 (8)	82.80 (19)	0.000	63.23 (18)	36.77 (9)	0.000	63.23 (18)	0.59 (1)	36.18 (8)	0.000	43.01 (13)	56.99 (14)	0.003
Chronic Bronchitis	81.43 (5)	18.57 (4)	0.001	100 (9)	0.00 (0)	0.111	100.00 (9)	0.00 (0)	0.00 (0)	0.056	81.42 (4)	18.58 (5)	0.001
Chronic Heart disease	27.39 (10)	72.61 (29)	0.000	97.19 (34)	2.81 (5)	0.000	97.19 (34)	0.00 (1)	2.81 (4)	0.000	59.51 (18)	40.49 (21)	0.000
Chronic Renal disease	48.02 (25)	51.98 (11)	0.581	99.6 (34)	0.40 (2)	0.000	99.60 (34)	0.00 (0)	0.40 (2)	0.013	49.71 (30)	50.29 (6)	0.499
Coronary Obstructive	35.45 (7)	64.55 (6)	0.000	100.00 (13)	0.00 (0)	0.001	100.00 (13)	0.00 (0)	0.00 (0)	0.003	59.57 (9)	40.43 (4)	0.000
Pulmonary disease													
Diabetes Mellitus	25.23 (65)	74.77 (122)	0.000	88.05 (168)	11.95 (19)	0.000	88.05 (168)	1.48 (6)	10.47 (13)	0.000	42.17 (94)	57.83 (93)	0.000
Gastrointestinal Disorder	31.33 (342)	68.67 (466)	0.000	94.67 (760)	5.33 (48)	0.000	94.67 (760)	3.05 (25)	2.28 (23)	0.000	47.75 (475)	52.25 (333)	0.000
High Cholesterol	22.89 (10)	77.11 (14)	0.000	75.94 (22)	24.06 (9)	0.000	75.94 (22)	24.06 (2)	0.00 (0)	0.000	46.95 (13)	53.05 (11)	0.000
Hypertension	32.39 (391)	67.61 (578)	0.000	94.64 (904)	5.36 (65)	0.000	94.64 (904)	3.32 (34)	2.04 (31)	0.000	50.90 (577)	49.10 (392)	0.000
Musculoskeletal Disorder	32.91 (120)	67.09 (255)	0.000	89.67 (344)	10.33 (31)	0.000	89.67 (344)	9.10 (20)	1.23 (11)	0.000	55.24 (188)	44.76 (187)	0.000
Neurological and Psychological Disorder	13.68 (14)	86.32 (28)	0.000	88.22 (39)	11.78 (3)	0.000	88.22 (39)	11.62 (2)	0.16 (1)	0.000	28.29 (19)	71.71 (23)	0.000
Skin disease	38.27 (76)	61.73 (113)	0.000	97.67 (183)	2.33 (6)	0.065	97.67 (183)	2.12 (4)	0.21 (2)	0.003	42.68 (101)	57.32 (88)	0.000
Stroke	34.43 (13)	65.57 (12)	0.000	93.35 (24)	6.65 (1)	0.000	93.35 (24)	6.65 (1)	0.00 (0)	0.000	25.07 (14)	74.93 (11)	0.000
Thyroid Disorder	13.11 (14)	86.89 (32)	0.000	94.23 (41)	5.77 (5)	0.000	94.23 (41)	0.00 (0)	5.77 (5)	0.000	83.73 (25)	16.27 (21)	0.000
Urinary Incontinence	20.72 (18)	79.28 (30)	0.000	99.96 (47)	0.04 (1)	0.000	99.96 (47)	0.00 (47)	0.04 (1)	0.000	34.65 (25)	65.35 (23)	0.000
Single morbidity	31.98 (1,145)	68.02 (1789)	0.000	93.28 (2,723)	6.72 (211)	0.000	93.28 (2,723)	4.31 (106)	2.41 (105)	0.000	49.44 (1,653)	50.56 (1,281)	0.000
Multimorbidity	18.49 (491)	81.51 (1,431)		86.68 (1,678)	13.32 (244)		86.68 (1,678)	6.06 (105)	7.26 (139)		37.96 (840)	62.04 (1,082)	

All diseases except chronic heart disease and skin disease significantly increased inpatient hospitalization. Diabetes (36.2%), neurological and psychological disorders (11.8%), asthma (11.1%), cancer (36.8%), and high cholesterol (24.1%) had the highest percent of inpatient hospitalization. The percent inpatient hospitalization was highest for individuals living with multimorbidity (13.3%) compared with those living with single morbidity (6.7%).

All diseases except chronic bronchitis and chronic renal disease significantly increased the percent duration of hospital stay. The percent of respondents having four or more days of hospital stay were highest for cancer (36.2%), diabetes (10.5%), and thyroid disorder (5.8%). The percent of respondents having four or more days of hospital stay were highest for individuals living with multimorbidity (7.26%) as compared with those affected with single morbidity (2.4%).

All diseases except chronic renal disease significantly increased the percent of outpatients visits. Stroke (74.9%), urinary incontinence (65.4%), diabetes (57.8%), skin disease (57.3%) and cancer (57.0%) had highest percent of outpatients’ visits. The percent outpatient visits were highest for individuals living with multimorbidity (62.0%) compared with those affected with single morbidity 50.6%).

Table 5 presents the variation in the OOPE for hospitalization and outpatient visits by selected NCDs among the aboriginal older adult population. The OOPE for hospitalization was highest for cancer (Rs. 16,592), thyroid disease (Rs. 4,736), chronic heart disease (Rs. 3,039), diabetes millets (Rs. 2,591), and hypertension (Rs. 1,388). The OOPE for hospitalization was highest for individuals living with multimorbidity (Rs. 1,971) compared with those affected with single morbidity (Rs. 1,242). Furthermore, Table 5 presents the OOPE for outpatients’ visits was highest for cancer (Rs. 3,313), neurological and psychological disorders (Rs. 2,703), stroke (Rs. 2,036), gastrointestinal disorders (Rs. 1,702), and urinary incontinence (Rs. 1,511). The OOPE for outpatient visits were highest for individuals living with multimorbidity (Rs. 1,535) compared with those affected with single morbidity (Rs. 1,242).

**TABLE 5:** Variations in OOPE (In INR) by non-communicable diseases among the aboriginal older adult population, Longitudinal Ageing Study in India (LASI), 2017–18.

Diseases	Hospitalization [Mean (S.E.)}			Out Patient Expenditure [Mean (S.E.)}		
	Total Expenditure	Reimbursed	Out of Pocket Expenditure	Total Expenditure	Reimbursed	Out of Pocket Expenditure
Asthma	1014 (429)	102 (45)	911 (399)	953 (232)	185 (59)	768 (183)
Cancer	17814 (7669)	1222 (931)	16592 (7598)	3609 (1546)	296 (139)	3313 (1457)
Chronic Bronchitis	0 (0)	0 (0)	0 (0)	732 (512)	222 (219)	510 (337)
Chronic Heart Disease	3685 (2581)	645 (645)	3039 (356)	1361 (541)	204 (98)	1156 (466)
Chronic Renal Disease	971 (711)	111 (77)	860 (638)	779 (423)	111 (77)	68 (368)
Coronary Obstructive Pulmonary Disease	0 (0)	0 (0)	0 (0)	742 (588)	0 (0)	742 (588)
Diabetes Mellitus	2633 (1536)	42 (21)	25908 (1531)	1417 (270)	245 (48)	1172 (230)
Gastrointestinal Disorder	1006 (259)	63 (12)	943 (253)	2105 (171)	403 (28)	1702 (156)
High Cholesterol	291 (251)	0 (0)	291 (251)	48 (41)	13 (83)	35 (15)
Hypertension	1457 (416)	69 (19)	1388 (413)	1402 (110)	264 (23)	1137 (96)
Musculoskeletal Disorder	829 (569)	23 (12)	805 (564)	1045 (135)	218 (32)	827 (11)
Neurological and Psychological Disorder	885 (699)	0 (0)	885 (699)	3083 (1129)	380 (122)	2702 (1055)
Skin Disease	156 (89)	10 (10)	145 (84)	1225 (211)	306 (52)	919 (172)
Stroke	0 (0)	0 (0)	0 (0)	2036 (2019)	0 (0)	2036 (2019)
Thyroid Disorder	4866 (3085)	130 (73)	4736 (3062)	1036 (415)	130 (73)	906 (358)
Urinary Incontinence	1 (1)	0 (0)	1 (1)	2009 (491)	499 (126)	1510 (372)
Single morbidity	1392 (220)	150 (86)	1241 (224)	1554 (73)	291 (13)	1262 (65)
Multimorbidity	2312 (467)	341 (125)	1970 (480)	1864 (116)	330 (17)	1534 (108)
Kruskal Wallis’ Chi square	20***	4***	16***	195***	67***	176***

Note: 1 USD = 65.09 INR, reserve bank of India, 2017.

*p*-value *p < 0.05, **p < 0.01, ***p < 0.001.

**FIGURE 1:**
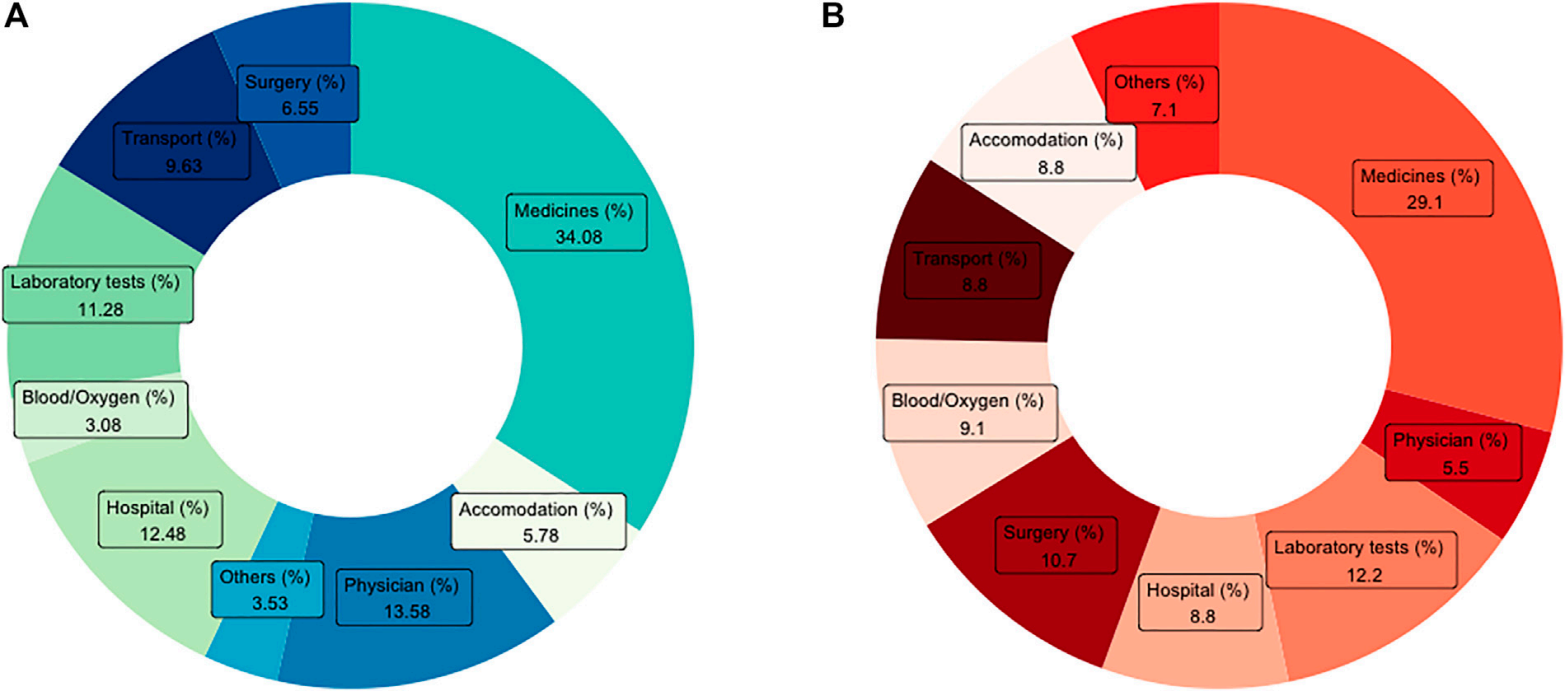
Distribution of **(A)** inpatient out of pocket expenditure (OOPE) and **(B)** outpatient OOPE for multimorbid aboriginal older adult population in India, longitudinal ageing study in India (LASI), 2017-18.

Figure 1 provides the distribution of OOPE expenditure into its components for hospitalization and out-patient visits among India’s multimorbid aboriginal older adult population. In case of hospitalization cases, the highest share of OOPE was spent on the medicines (34.1%), followed by the physician’s fees (13.6%), hospital’s fees (12.5%), laboratory tests (11.3%), and transportation cost (9.6%). For outpatient visits, the highest share of OOPE was spent on medicines (29.1%), followed by laboratory tests (12.2%), surgery (10.7%), blood/oxygen (9.1%), and hospital charges (8.8%).

## Discussion

Despite rapid strides in health care improvement and socio-economic development in India, the aboriginal population, also known as the scheduled tribes (tribal) remains one of the most vulnerable sub-group in the country. There is ample evidence indicating a perpetual disparity in health and nutrition outcomes among tribal population vis-à-vis their non-ST counterparts (this includes all the social caste groups other than the Scheduled Tribes) (5). Existing studies have focussed on exploring various health realms for this population, which are not limited to malnutrition (9), risky health behaviours (14, 15), infectious (14, 16), and non-communicable diseases burden (14, 16).

Commensurate with changing lifestyle and living condition, the tribal population are witnessing a demographic and health transition with an increasing non-communicable disease burden. There is a need to garner empirical evidence on the non-communicable disease profile and its implications on the aboriginal older adult population at the national level. Most of the studies conducted are based on smaller samples or specific regions in the country (9, 10). These studies warrant an urgent need to generate evidence about the aboriginal population at the national level. Thus, the study utilizes data on 11,365 aboriginal individuals aged 45 years and above from LASI, 2017-18; and presents compelling evidence on the burden and correlates of multimorbidity. Furthermore, the study explores the linkages of multimorbidity with increased healthcare utilization and OOPE.

Findings suggest that twenty-seven and fifteen percent of the aboriginal older adult population was affected with single disease and multiple morbidity, respectively. The burden of single morbidity is similar to that reported at the national level (17). However, the prevalence of multimorbidity was lower for the aboriginal population than the national average for older adults (17). The estimates for multimorbidity were also lower than that reported in other studies (18, 19). As the data used in the study is self-reported, the estimates generated to comply with the opportunistic screening, are pretty low among the aboriginal groups, given their socio-economic backdrop in India (20). Aiming at improved prevention, early detection, and effective management, the government initiated various NCD control programs in India (21). However, as the aboriginal population usually resides in remote locations, they have inadequate awareness of risky health behaviours and non-communicable diseases (4, 14). Additional issues like poor health care delivery system, scepticism, and myths reducing the utilization of healthcare infrastructure, and poor affordability are major precursors to low healthcare accessibility among the aboriginal population (4, 14). These pretexts act as a catalyst in worsening the chances of universal health access.

Hypertension, gastrointestinal disorders, musculoskeletal disorders, and diabetes were the most prevalent NCDs among aboriginal older adults. Similar NCDs are reported to hold a higher burden among the older adult population in the country (17). Other studies conducted in India also reported preponderance of hypertension (17, 19, 22, 23), a range of musculoskeletal disorders (17, 19, 22), gastrointestinal disorders, and diabetes (17, 19, 23) in India.

In addition, individuals in the higher age range were more likely to report multimorbidity. Similar findings were reported by various studies in India (17, 24–27). Multimorbidity can be understood as a result of the age-induced physiological breakdown, coupled with generic responses caused by biological stress engendered by long-term exposure to important life events and other environmental factors (28).

Respondents with higher education and wealth reported a higher prevalence level of multimorbidity. These findings highlight that burden was higher for individuals with better access to the healthcare delivery system and health information. Also, individuals from the affluent classes have better chances to afford healthcare facilities (17, 18, 29). Another possible explanation is that educated individuals have better health-seeking behaviours and are less likely to be influenced by the existing myths regarding health or healthcare in their community (17). Better accessibility, affordability and an adequate level of health-seeking behaviour can lead to achieving the sustainable development goals of health for all.

Currently unemployed individuals were more likely to report multimorbidity. Existing literature has highlighted the relationship between employment and various NCDs (17, 30, 31). In the existing literature for multimorbidity in India, even recent studies reported that the burden of multimorbidity was higher for the older adults who are not currently working (17, 32–34). Currently unemployed individuals include individuals who never worked or those who have already retired from the workforce. In both of these cases, individuals might be more likely to confront stress induced by economic instability and income insecurity (31, 35, 36), which could lead to chronic health conditions like diabetes, hypertension, asthma, cancer, anxiety, gastrointestinal issues, and heart disease (17, 35–37).

Furthermore, the findings suggest that those living with multimorbidity hold a disproportionally higher health care utilization burden than their counterparts. The percent visiting healthcare facility was 14 percentage points higher for the aboriginal population living with multimorbidity than those with single morbidity. Similarly, the percent of inpatient visits, duration of stay, and percent outpatient visits were 6.6, 2.4, and 11.5 percentage points higher for those living with multimorbidity than those with single morbidity, respectively. Similar findings were also reported by studies based on small sample sizes in India, which reported that utilization of healthcare services would increase substantially if an individual simultaneously suffers from multiple diseases (32, 38).

The results suggest that multimorbidity is associated with a higher burden of OOPE for both hospitalization and outpatient visits than only single disease. The mean OOPE on hospitalization and outpatient visits was 1.6 and 1.2 times higher for patients with multimorbidity than for those with single morbidity. Existing literature in the domain reported that expenditure incurred by multimorbidity was three times higher than single morbidity (32, 38). The highest expenditure on hospitalization was incurred by cancer, followed by the thyroid and chronic heart disease. Cancer was also reported as the most expensive disease, followed by neurological and psychiatric disease, and stroke considering the OOPE on outpatient visits. It is worth mentioning that during hospitalization, the highest share of burden was contributed by medicines, followed by physician’s consultation fees and hospital charges. In contrast, the highest share of the burden for outpatient visits was contributed by medicines, followed by laboratory tests.

The primary strength of the study is the use of comprehensive large-scale nationally representative data on older adults. The study presents novel evidence on the burden and correlates of multimorbidity among India’s aboriginal older adult population. In addition, the study explores the linkages between multimorbidity and four indicators of health care utilization, namely visited health facility/healthcare provider, number of inpatient hospitalizations, duration of stay, and number of outpatients visit. Furthermore, the association between OOPE incurred during hospitalization and outpatient visits was examined. In addition to this, the percent share of OOPE on each item was computed. The study identifies diseases that incur the maximum burden of health care utilization and expenditure for the aboriginal older adult population in the country. Despite all these strengths, the NCDs used are self-reported, resulting in misclassification bias. Also, as secondary cross-sectional data was employed, the study assessed no causality.

Our study provides vital insights into the non-communicable disease scenario for India’s aboriginal older adult population. Findings necessitate strategies to combat the accelerating multimorbidity burden among this vulnerable section of society. Government should extend population-based awareness and screening programs to ensure better disease reporting for the most disadvantaged sub-groups among the aboriginal population. Initiating and extending strategies to strengthen the existing healthcare delivery system in the form of Health and Wellness Centres (HWCs) in the remotely located tribal regions can help to combat the challenges posed by multimorbidity in the country. Revolutionizing primary health centres and HWCs into a more patient-oriented set-up, upholding health care expenditure at a minimum is warranted. Particular emphasis needs to be laid on the cost incurred by the medicines, consultation fees and hospital charges, ensuring an equal chance of good health and well-being for the most disadvantaged population in India.

Future research should focus on executing similar studies exploring the extent of multimorbidity and its impact on healthcare utilization, health care expenditure, and distress financing, comparing various social groups in India. Also, studies need to be done to explore the extent of satisfaction felt during hospitalization and outpatient visits by the aboriginal older adult population in the country. Replicating our study findings using a cohort-based set-up for the aboriginal population is necessitated.

## Data Availability

The study utilised a de-identified data from a secondary data source, Longitudinal Ageing Study in India (LASI), 2017-18. The data has been archived in the public repository of LASI held at the International Institute for Population Sciences, Mumbai. The access to the data requires registration which is granted specifically for legitimate research purposes. Requests to access the data should be made to datacenter@iipsindia.ac.in.
